# Brucellosis detection and the role of *Brucella* spp. cell wall proteins

**DOI:** 10.14202/vetworld.2023.1390-1399

**Published:** 2023-07-04

**Authors:** Aitbay Bulashev, Saule Eskendirova

**Affiliations:** 1 Department of Microbiology and Biotechnology, S. Seifullin Kazakh Agrotechnical Research University, Astana, Kazakhstan; 2 Laboratory of Stem Cell, National Center for Biotechnology, Astana, Kazakhstan

**Keywords:** *Brucella*, diagnostics, enzyme-linked immunosorbent assay, outer membrane protein, periplasmic protein

## Abstract

Brucellosis remains an endemic zoonotic disease in many developing countries, causing great harm to public health and devastating losses to livestock. One of the main reasons for the low effectiveness of anti-brucellosis measures is the lack of reliable methods for diagnosing infected animals throughout their lifespan. Classical serological tests, such as the tube agglutination test, rose Bengal plate test, and complement fixation test, as well as commercial enzyme-linked immunosorbent assay kits, are based on the detection of antibodies to the cell wall polysaccharide antigens of *Brucella* spp. smooth strains. As a result, they do not exclude cross-reactions with related bacteria and fail to differentiate between infected and vaccinated animals. Over the past decades, many attempts have been made to identify immunoreactive and pathogen-specific protein antigens. To date, several studies have investigated *Brucella* spp. recombinant proteins, including cell wall proteins, as the best antigens for diagnosing brucellosis in animals and humans. However, the available results on the specificity and sensitivity of serological tests based on cell wall proteins are ambiguous and sometimes contradictory. This review aims to provide an overview of the current state of knowledge of the diagnostic value of outer membrane and/or periplasmic proteins of *Brucella* spp. The goal is to identify future developments that may lead to reliable antigens for serological tests.

## Introduction

The key element in the brucellosis elimination measures is the timely isolation of infected animals. Serological tests developed at the beginning and/or in the second half of the last century, such as the tube agglutination test (TAT), complement fixation test (CFT), and rose Bengal plate test (RBT), remain the main methods of intravital diagnostics to this day [1]. These conventional tests, as well as currently available enzyme-linked immunosorbent assay (ELISA) kits, are based on the detection of antibodies against the cell wall lipopolysaccharides of smooth strains (S-LPS) of *Brucella* spp., which does not exclude cross-reactions with related bacteria [2, 3]. It is worth noting here that the introduction of *Brucella* S-LPS based commercial ELISA kits into the diagnostic practice of the Republic of Kazakhstan (2008–2013) was unsuccessful, as the number of animals testing positive for brucellosis increased by several times, and the epizootic situation did not improve [4]. Thus, practical experience has shown that ELISA, as one of the highly sensitive tests, could only be used in the “test and slaughter” strategy in the presence of a pathogen-specific antigen.

Over the past few decades, *Brucella* cell wall proteins screened with S-LPS have become the focus of study as promising immunogens for vaccine development and as components for creating specific diagnostic antigens. This review aims to summarize and analyze the current state of knowledge on the serological potential of *Brucella* spp. outer membrane and periplasmic proteins and to identify promising studies that can improve the diagnosis of brucellosis.

## Reactivity and Specificity of Native *Brucella* Cell Wall Proteins

The cell wall of *Brucella* consists of a thin peptidoglycan layer tightly bound to the outer membrane, in which three groups of proteins have been identified. These groups include the major outer membrane proteins (Omps) of Group 2 (porin, 36–38 kDa), Group 3 (25–27 kDa) [5], and the minor Omps of Group 1 (<92 kDa) [6]. In addition, Omps with molecular weights (MW) of 10, 16, and 19 kDa, exposed on the cell surface, have been identified as lipoproteins [7]. The genes encoding Group 2 porin proteins consist of two segments, Omp2a and Omp2b, which are closely linked in the *Brucella* genome and share a great degree of identity (>85%) [8].

Another *Brucella* spp. cell wall protein is BP26 (also known as Omp28) and Cu/Zn superoxide dismutase (SOD). BP26 was independently described by three scientific groups as a potential diagnostic antigen for brucellosis serodiagnosis [9–11]. It is located in the periplasmic space of the cell wall and functions as a transmembrane receptor. BP26 is a highly conserved protein for all *Brucella* species [11]. However, there is still no consensus regarding its localization. According to Lindler *et al*. [10], BP26 is located in the outer membrane and vesicles, while Cloeckaert *et al*. [9] found this protein inside cells as a soluble protein, using monoclonal antibodies (mAb).

*Brucella* spp. SOD is located in the periplasmic space of the cell wall and is a metalloenzyme that catalyzes the dismutation of superoxide ions. It is a key factor in protecting the pathogen from the respiratory burst of phagocytic host cells, helping it to survive and proliferate in phagocytes [12]. Recently, SOD has been shown to act as a VirB-independent type IV secretion system effector during *Brucella* infection [13].

*Brucella* cell wall proteins are of great interest to researchers looking for a non-polysaccharide antigen for serological diagnosis of the disease. Over the past few decades, many attempts have been made to identify antigenic and pathogen-specific proteins. Chin reported that high titers of antibodies against *Brucella ovis* intact cells were observed in both naturally infected and vaccinated rams using an indirect ELISA (i-ELISA), while in the case of using LPS as an antigen, antibodies from vaccinated animals showed significant activity. The extracts of the outer membrane complex bound well to antibodies from naturally infected rams, while sera from inoculated animals gave positive results only in initial sera dilutions [14].

Salt-extractable proteins fractionated by differential ammonium sulfate precipitation were used in immunoblotting to detect bovine immunoglobulin G (IgG) antibodies to *Brucella*. Antibodies of infected cows (Group 1) and cows vaccinated with *Brucella abortus* 19 and then subsequently infected with brucellosis (Group 2) reacted with soluble proteins with MW from 31 to 45 kDa. Immunoglobulins of animals in the second group additionally revealed protein fractions with MW ranging from 66 to 71 kDa. Immunoglobulin G antibodies from healthy cattle and vaccinated calves did not bind to any protein groups [15].

Connolly *et al*. [16] identified 163 proteins in the cell wall of *B. abortus* cell envelope using 2D electrophoresis (2-DE) with matrix laser desorption/ionization, time-of-flight mass spectrometry, and liquid chromatography-tandem mass spectrometry (MS/MS). These included cell wall proteins such as Omp25, Omp31, and Omp2b. Immunoblotting analysis using *Brucella*-infected bovine and/or human antisera revealed several novel immunoreactive proteins in the *B. abortus* cell wall.

Al Dahouk *et al*. [17], using 2-DE and immunoblotting, identified SOD as one of the 17 most immunogenic *Brucella* proteins suitable for serological analysis. It was found in three types of antigens used: Native antigen, standard agglutination, and commercially available agglutination antigens. Protein cross-reactivity was negligible. The surface-enhanced laser desorption/ionization mass spectrometry spectra also clearly distinguished *B. abortus* from related bacteria.

According to Pajuaba *et al*. [18], one-dimensional electrophoretic profiles of *B. abortus* S19 antigen, obtained using Triton X-114, showed several polypeptides with MWs ranging from 10 to 79 kDa. Three proteins with MWs of 10, 12, and 17 kDa were recognized only by antibodies of naturally infected cows (n = 30) and did not bind to the sera of heifers vaccinated with *B. abortus* S19 (n = 30). The authors suggest that these proteins could be used as novel antigens for differentiating infected from vaccinated animals (DIVA).

The immunoreactivity of the 2-DE separated proteins was determined by immunoblotting using antisera from cattle infected with *B. abortus* and/or *Yersinia enterocolitica*. Individual proteins binding to positive anti-*B. abortus* sera were identified by MS/MS analysis. Eighteen *B. abortus* 1119-3 proteins, including the periplasmic proteins Omp28 and SOD, showed immunoreactivity only against homologous positive antisera. The isolated immunodominant proteins are considered antigens to avoid cross-reaction in the diagnosis of brucellosis [19].

Kim *et al*. [20] reported the immunogenicity of *B. abortus* RB51 proteins separated by 2-DE and subjected to immunoblotting using four types of antisera: *Brucella abortus*, *Y. enterocolitica* O:9, *Escherichia coli* O157:H7, and negative bovine sera. The periplasmic SOD is one of 11 highly immunoreactive proteins that may be useful as alternative antigens to reduce cross-reactivity.

Falcão *et al*. [21] evaluated antibody responses in seropositive cattle with brucellosis and seronegative vaccinated animals, targeting protein bands in western blotting. The most antigenic band in the western blotting, which accurately distinguished seropositive from vaccinated cattle, had an MW of ≤20 kDa. The authors propose that western blotting could be used as a confirmatory test for diagnosing bovine brucellosis.

Faria *et al*. [22] described the immunoproteomic profile of *B. abortus* 2308 by 2-DE western blotting using a pool of sera from cattle vaccinated with S19 and/or RB51, naturally infected with brucellosis, and unvaccinated seronegative animals. Protein evaluation using three groups of sera showed the antigenicity of SOD for antibodies from infected animals. Another study noted a significant increase in SOD activity in seropositive pregnant cows (n = 10) compared with seronegative analogs (n = 10) [23].

## Serological Potential of *Brucella* spp. Recombinant Omps (rOmps)

Among the major Omps of *Brucella* spp., Omp25 and Omp31 have been studied in terms of their antigenic and immunogenic properties. Omp25 is highly conserved in different *Brucella* species, while Omp31 is present in all species except *B. abortus* [24]. However, a *B. abortus* membrane protein with an apparent MW of 31 kDa (Omp31b) has been described; it possesses some similarity to Omp31 from other strains of *Brucella* [25]. A comparison of the nucleotide sequence of *B. ovis* Omp31 with that of Omp31b showed 77% identity [26].

Recombinant DNA technology is an effective method for producing numerous proteins in a short time, which is fast and safe. Recombinant Omp31 is the first *Brucella* protein cloned and expressed in *E. coli*. Antiserum against rOmp31 detected the protein in western blotting in 34 *Brucella* strains of all six main species. The natural analog of the recombinant protein was present in all eight *B. abortus* biovars tested [27].

The antigenicity of *Brucella melitensis* rOmp31 was studied in an i-ELISA using human sera, as well as sera from animals with positive and negative results for brucellosis according to classical serological tests. Brucellosis was bacteriologically confirmed in all sheep and dogs and in 42% of human cases of brucellosis. Specific antibodies to the pathogen were detected in 48%, 61%, and 87% of infected humans, sheep, and dogs, respectively. According to the authors, rOmp31 “would be of limited value for the diagnosis of human and animal brucellosis,” but it could be used in combination with other recombinant proteins [28].

Recombinant Omp31 from *B. ovis* was used in an i-ELISA for testing bovine sera (n = 10) that were positive to culture and fluorescence polarization assay (FPA), as well as negative sera (n = 10) to RBT, FPA, and microbiological tests. The results not only showed the antigenicity of the protein but also demonstrated reliable differences between the optical density (OD) values of positive and negative sera, with the highest readings found in positive sera [26]. Rosales *et al*. [29] also described sufficient sensitivity (77%) and high specificity (91%) of an i-ELISA based on rOmp31 from *B. melitensis* 16M in serological testing of cattle.

We conducted a study to compare the antigenicity of *B. melitensis* rOmp31, *B. abortus* rOmp25, and native soluble protein antigen (SPA) of both species on cattle blood serum samples with positive results for brucellosis by TAT and CFT. An i-ELISA based on SPA of *B. melitensis* and/or *B. abortus* showed the presence of antibodies in 68% and 60% of seropositive cattle, respectively. The use of rOmp25 and rOmp31 as antigens in the immunoassay confirmed the presence of specific antibodies in only 52% and 36% of seropositive animals, respectively. These results suggest a higher specificity of the SPA and rOmps than that of S-LPS, the main antigen in conventional tests. It should be noted that *Brucella* SPA also contains cell wall polysaccharides, which could interact with antibodies formed against S-LPS of related bacteria. The relatively low sensitivity of i-ELISA/rOmp25 or i-ELISA/rOmp31 compared to that of conventional tests and i-ELISA/SPA seems to indicate a higher specificity of the immunoassay variants based on rOmps [30, 31].

The serological potential of *B. abortus* rOmp2a was evaluated using clinical sera from patients with (i) brucellosis-positive RBT and TAT, (ii) bacteriologically confirmed diagnosis, and (iii) negative serological tests. In addition, blood sera from patients with other febrile diseases and healthy donors were used as controls. The sensitivity and specificity of i-ELISA/rOmp2a and western blotting were very high (94%–96%) [32]. Sufficient specificity was also established for rOmp2b: sera from mice inoculated with *E. coli* *O:157*, *Salmonella enterica*, and *Y. enterocolitica* O:9 did not react significantly with rOmp2b in an i-ELISA. Analysis of cattle blood sera (n = 250) using i-ELISA/rOmp2b compared with RBT and TAT showed that 12% of sera positive for conventional tests were found to be negative for the immunoassay, while none of the sera from healthy cattle showed a false-positive result for i-ELISA/rOmp2b [33].

The antigenicity of proteins with MW of 10, 16, and 19 kDa and the possibility of using them in brucellosis serodiagnosis is still poorly understood. They are mostly studied not for their antigenic properties but for their immunogenic and/or protective properties, both as a single immunogen [34] and along with other proteins [35, 36]. A vector vaccine against animal brucellosis based on Omp16 and L7/L12 proteins has already been introduced into the practice of veterinary medicine in the Republic of Kazakhstan [37].

According to Tibor *et al*. [38], most of the sera from naturally infected sheep were reactive to *B. abortus* rOmp10 and rOmp19 in an i-ELISA. In contrast, sera from infected cattle were almost completely unreactive. The presence of both proteins in the 34 strains tested, representing the most common *Brucella* species and all their biovars, was confirmed by immunoblotting with anti-rOmp10 and anti-rOmp19 mAbs. The authors concluded that host humoral responses may differ between animal species and/or the infecting *Brucella* strains.

Letesson *et al*. [39] studied the diagnostic value of rOmp10, rOmp16, and rOmp19, as well as major rOmp36 and rOmp25, in experimentally infected cattle, sheep, and goats. They established a delayed response of the immune system to the proteins of the pathogen compared to its S-LPS antigen. Interestingly, none of the five rOmps detected experimentally infected pregnant cows, pregnant ewes, or naturally infected cattle from brucellosis-free regions. Antiprotein antibodies were found only in experimentally infected pregnant animals kept in brucellosis-endemic regions. The authors explained the antibody response against the used protein in animals from the brucellosis-affected region through vaccination and concluded that ELISA based on *Brucella* proteins cannot be used in areas with a low prevalence of brucellosis. This fact, in our opinion, may also be due to the phenomenon of immunizing subinfection [40], where immunity develops in animals from a brucellosis-affected herd even without vaccination because of long-term and latent immunization of the body with small doses of the pathogen that are not capable of causing disease.

An i-ELISA using *B. abortus* rOmp16 was tested to detect anti-*Brucella* antibodies in human serum samples (n = 70). The results were assessed using commercial IgG ELISA kit and RBT. The diagnostic efficiency of ELISA/rOmp16 compared to that of RBT showed high sensitivity (100%) and specificity (95%), and complete agreement between the results of the test and the commercial ELISA kit was observed [41].

Polyclonal rabbit antibodies against *B. abortus* rOmp19 were used in an immunofluorescent test to identify the homologous pathogen species. The FITC-conjugated anti-rOmp19 antibody accurately recognized *B. abortus* cells and did not bind to *E. coli*, *Salmonella*, and *Klebsiella*. However, the test failed to recognize all *Brucella* isolates, and it showed positive results when testing half of the isolates of related bacteria used, which is likely due to the presence of common epitopes with heterogeneous microorganisms [42].

In our studies, we investigated the reactivity of *B. abortus* rOmp19 compared with the two main proteins, *B. abortus* rOmp25 and *B. melitensis* rOmp31, in blood sera from cattle that were experimentally infected with virulent *B. abortus* 544 [43]. We confirmed the specificity of the proteins expressed by *E. coli* BL21 (DE3) using antibodies from a rabbit that was immunized with phenol-inactivated *B. abortus* 19 cells. Antibodies against rOmp19 were detected in all experimentally infected animals (n = 12) as early as 14 days post-infection (p.i.), while antibodies to rOmp25 were only detected on the 28^th^ day p.i. By this time, antibodies against rOmp31 had not yet been detected in 25% of the animals. Furthermore, on the 28^th^ day p.i., antibody titers against rOmp19 were significantly higher than those against the other two proteins. These results suggest that Omp19 is more antigenic than the major proteins of the third group. Our findings are consistent with those of other studies that have found that antibody responses to minor proteins, including Omp19, are somewhat stronger than those to major proteins. This may be due to the location of Omp19, which is a surface lipoprotein and not an integral membrane protein [39].

The reactivity to post-vaccination (p.v.) and/or p.i. antibodies is the main criterion for evaluating the diagnostic value of recombinant proteins. In a mouse model, it was found that an i-ELISA based on a combination of three rOmps (rOmp25, rOmp28, and rOmp31) can differentiate p.i. antibodies to *B. melitensis* from those associated with vaccines or cross-reactivity with *Y. enterocolitica* O:9 [44]. However, in our study, we detected antibodies specific to rOmp25 and rOmp31 in more than half of the cattle kept in a brucellosis-free herd within 10 months after revaccination with *B. abortus* 19 [45]. This finding suggests that the results obtained in mice may not always apply to productive animals. We suggest that Omps can be used in an i-ELISA to test for brucellosis only in non-immune livestock before vaccination or in vaccinated animals after a certain time, which depends on the type of vaccines.

## The use of Recombinant *Brucella* spp. Periplasmic Proteins for Diagnosis of Brucellosis

Cloeckaert *et al*. [46] evaluated the antibody responses of sheep infected with *B. melitensis* H38 and *B. melitensis* Rev.1-vaccinated to the periplasmic protein rBP26 using an i-ELISA. Although the specificity and sensitivity of the immunoassay were quite high compared to traditional tests for diagnosing brucellosis, the OD of i-ELISA/rBP26 did not reach the values observed in an i-ELISA based on purified O-polysaccharide (O-PS). It should be noted that in experimentally infected sheep, the antibody response to rBP26 was delayed and much weaker than that to O-PS, and no antibody response against rBP26 was detected in vaccinated sheep. In our study, the OD values of an i-ELISA based on another native antigen, *Brucella* spp. SPA, were also higher than those of an i-ELISA/rBP26 in the serological investigation of cattle for brucellosis [47]. These differences can be explained by the lower specificity of the native O-PS and/or SPA antigens compared to the recombinant protein. Nevertheless, Kumar *et al*. [48] reported the superiority of *B. melitensis* rOmp28 over native antigens such as cell envelope antigen and whole cell sonicated antigen in the serological diagnosis of ovine and caprine brucellosis. This inconsistency is likely because of the use of different methodologies for obtaining native antigens and/or conventional serological tests as a standard for assessing the diagnostic value of the recombinant protein.

Seco-Mediavilla *et al*. [49] sequenced the gene encoding BP26 of reference strains of *B. abortus*, *Brucella suis*, *B. ovis*, as well as vaccine strains of *B. abortus* S19 and *B. abortus* RB51. Minor nucleotide substitutions were observed between the tested strains, with no modification of the amino acid sequence. They mapped BP26 epitopes using mAbs and recombinant DNA techniques and identified an immunodominant region of the protein for serodiagnosis of *B. melitensis* and *B. ovis* infections. The engineered recombinant fusion protein was not inferior in antigenicity to the whole rBP26 in immunoblotting using sera from sheep naturally infected with *B. melitensis* or *B. ovis*. Moreover, the fusion protein, unlike the whole protein, did not give false-positive results with sera from healthy sheep.

Gupta *et al*. [50] identified rBP26 as an immunodominant protein for detecting infection in cattle, sheep, goats, and humans and proposed using it as an informative antigen to study the humoral response of animals infected with brucellosis. The results of ELISA/rBP26-positive animals were compared and evaluated by polymerase chain reaction using the *B. melitensis* BP26 gene as the target, and a complete positive correlation was established between the immunoassay and the molecular method.

The immunodominant region of BP26, with an MW of 10 kDa, was used as an antigen in ani-ELISA for screening bovine serum samples from three groups: (i) presumptively negative animals (n = 70), (ii) random samples from brucellosis-affected herds (n = 308), and (iii) calves vaccinated with *B. abortus* S19 (n = 30). Analysis of presumptively negative samples showed 100% and 98% ELISA specificity compared to RBT and TAT, respectively. Among random samples from brucellosis-affected herds, the coincidence of the results of an i-ELISA and classical tests was within 78%–81%. All sera from vaccinated animals (n = 30) taken between 25- and 35-day post-vaccination (p.v.) with *B. abortus* S19 were antibody-free by i-ELISA/rBP26, while RBT and TAT resulted in 30% and 97% false positives, respectively. It has been suggested that an i-ELISA based on truncated rBP26 may find its application in DIVA [51].

The diagnostic value of *B. melitensis* rOmp28-based ani-ELISA was studied on human serum samples in its plate and dot versions compared with RBT and TAT. The periplasmic protein bound only to antibodies of sera obtained from patients with a bacteriologically confirmed diagnosis and was not recognized by the sera of culture-negative patients. Both plate and dot versions of the i-ELISA had a correlation of 90% with conventional tests. The sensitivity of plate i-ELISA was higher (98%) than that of dot-ELISA (82%), but the latter had higher specificity (92% vs. 86%) [52]. In another study, patient samples collected from hospitals (n = 60) and samples from healthy donors (n = 30) were tested using an i-ELISA based on the rOmp28 precursor protein of *B. melitensis* Rev1 versus RBT. The recombinant antigen-based immunoassay was successful in terms of sensitivity, specificity, and positive/negative predictive values [53].

Tian *et al*. [54] reported good reactivity of BP26 to anti-*Brucella* bovine sera but did not rule out the possibility of obtaining false-positive results when using it as an antigen. In a western blot study of 44 bovine sera, BP26 interacted with antibodies from 30 seropositive animals but showed false-positive results with the rest of the sera. Moreover, truncated fragments of the rBP26 could not exclude false-positive results in the detection of *Brucella*-free sera. It should be noted here that the diagnostic value of rBP26 was compared only to i-ELISA/LPS, so serologically negative cattle may be positive for *Brucella*.

Bai *et al*. [55] also used LPS as well as rose Bengal antigen as positive controls in studying the suitability of rBP26 and 5 rOmps (MWs of 10, 16, 19, 25, 31 kDa) as an i-ELISA antigen in testing human and goat sera but obtained more encouraging results. RBP26 showed the highest diagnostic accuracy of 96% and 95%, respectively, while rOmp31 was more accurate in diagnosing bovine sera (84%). These contradictions seem to support the specificity of rBP26 not only to hosts but also to the *Brucella* species previously described by Xin *et al*. [56] when studying antibody production against rBP26 in sheep (n = 15), goats (n = 15), and cattle (n = 6) during experimental infection with various species of *Brucella* using LPS and/or rBP26-based i-ELISA. The results showed that all infected animals could produce antibodies with high titers against LPS; however, only sheep infected with *B. melitensis* 16M and *B. melitensis* M28 and goats infected with *B. melitensis* 16M and *B. abortus* 2308 could produce antibodies against BP26. However, in the sera of sheep and goats infected with *B. suis* S1330 and sheep and cattle infected with *B. abortus* 2308, antibodies against rBP26 were not detected. Serological testing of naturally infected animals also confirmed that i-ELISA/rBP26 cannot detect all infected animals. The phenomenon of rBP26 is bacterial and host-specific, limiting its reliability as a substitute for LPS used in a conventional ELISA. Previously, the absence of a humoral immune response to Omp28 in cattle and pigs was described by Lindler *et al*. [10].

The periplasmic protein SOD is less studied in terms of diagnostic value than BP26. It catalyzes the conversion of the superoxide radical to hydrogen peroxide. As an antioxidant enzyme, it regulates the level of O_2_ in the cell and plays an important role in the death of phagocytosed bacteria [57]. Therefore, it is no coincidence that *Brucella* SOD is considered by researchers to be a promising protein in the creation of new generation vaccines [58], and thus its diagnostic value remains insufficiently studied.

The SOD protein was detected in all *Brucella* species and biovars used, including eight biovars of *B. abortus*, except for *Brucella neotomae* and biovar 2 of *B. suis*, by western blotting using rabbit antiserum against the recombinant analog [59]. The antigenicity of recombinant SOD (rSOD) was studied in an i-ELISA on blood serum samples of dogs with known positive (n = 30) and negative results (n = 194) by TAT. rSOD conferred 100% sensitivity and 99% specificity to the test [60].

Faria *et al*. [22] used a pool of bovine sera from (i) cattle vaccinated with *B. abortus* S19 and/or RB51, (ii) those naturally infected with brucellosis, and (iii) unvaccinated seronegative animals. They proved the possibility of using rSOD and malate dehydrogenase as a reliable antigen for diagnosing bovine brucellosis and DIVA in the early post-vaccination stages.

Nagalingam *et al*. [61] reported the results of an antigenicity study of 10 recombinant *B. abortus* proteins by western blotting and i-ELISA using positive and negative bovine serum samples (n = 113 each). Six proteins, including rSOD and rBP26, showed a reaction with brucellosis-positive bovine serum, whereas none of the proteins were recognized by rabbit anti-*Y. enterocolitica* O:9 antibodies. The authors believe that rBP26 could be used as a diagnostic antigen to exclude *Yersinia* infection in the diagnosis of cattle brucellosis.

We conducted a comparative study of the serological potential of the outer membrane (rOmp25 and rOmp31) and periplasmic proteins (rSOD and rBP26). The former proved to be more informative in detecting antibodies in cattle from a new focus (outbreak) of brucellosis infection (n = 77), while the latter significantly surpassed them in antigenicity in the study of blood sera from animals kept in a brucellosis-affected (endemic) farm (n = 43). Differences in the antigenicity of rOmps and periplasmic proteins for these two groups of cattle are associated with the localization of the proteins in the *Brucella* cell. Omp25 and Omp31 are surface proteins, while SOD and BP26 are located between the peptidoglycan and the inner membrane of the cell wall, and therefore less accessible to the immune system than the outer membrane structure [62]. In all likelihood, in cattle of a fresh infection focus, antibody formation primarily develops against Omps. The immune response to “deep” proteins occurs later, when the pathogen overproduces SOD to detoxify the radicals generated by the host’s antimicrobial response, which occurs in animals of a brucellosis-affected herd. In this regard, we have expressed the possibility of using rOmps for i-ELISA screening of cattle in brucellosis-free areas for early detection of infected animals, and periplasmic proteins for scheduled serological testing of animals in a brucellosis-affected farm.

In our subsequent study of cattle kept in a brucellosis-free herd (n = 48), antibodies to rSOD were not detected at 10 months post-vaccination with *B. abortus*19S. However, some animals reacted positively by an i-ELISA based on rOmps19, 25, 31, and rBP26 [63].

Recently, a conserved periplasmic protein named EipA has been reported, which plays a role in maintaining the integrity of the *Brucella* spp. cell wall and influences the survival of the pathogen in host cells [64]. However, the immunoreactivity of the protein remains unexplored.

## Using Combined and Multi-epitope Proteins in Improving the Diagnosis of Brucellosis

From a practical point of view, the use of a single protein in immunoassays cannot ensure the detection of the entire population of pathogen-specific antibodies. Therefore, reliable diagnostic results can be achieved when multiple recombinant proteins are used simultaneously.

Simborio *et al*. [65] compared the diagnostic value of *B. abortus* rOmp10, rOmp19, and rOmp28, both individually and in combination, as antigens in an i-ELISA using TAT-positive and TAT-negative cattle serum samples. The combined rOmps showed greater reactivity than single proteins in positive sera. A study was conducted to determine the diagnostic value of various combinations of rBP26 with five rOmps (MW of 10, 16, 19, 25, and 31 kDa). Sera from rabbits infected with related bacteria were used to test the specificity of the combined antigens. Efficacy analysis was performed by an i-ELISA on human (n = 161), goat (n = 120), and bovine sera (n = 144) with positive and/or negative TAT and RBT results. The protein combination of rOmp25 + rOmp31 + rBP26 showed the desired efficiency for the detection of *Brucella*-specific antibodies in human sera, while rOmp31/BP26 proved to be the best combination for serological testing of goat and cattle serum samples. Cross-reaction analysis showed that the protein combinations used did not respond to antibodies against common pathogens [66].

An i-ELISA based on 3 rOmps (MW of 25, 28, and 31 kDa) made it possible not only to bypass the effects of cross-reacting antibodies but also to differentiate mice infected with *B. melitensis* from vaccinated analogs [44]. In serological examinations of human sera, the combined antigen consisting of rOmp2b, rOmp31, and rBP26 was more effective than single proteins in an i-ELISA. The specificity of the proteins used was higher than that of LPS because they did not cross-react with *Y. enterocolitica* O9 and *E. coli* O157:H7 [67].

Comprehensive studies have been conducted on the construction of *Brucella* spp. multi-epitope antigens over the past 2–3 years. Yin *et al*. [68] reported that an antigen consisting of the determinants rOmp16, rOmp31, rOmp2b, and rBP26 correctly identified positive and negative goat serum samples. The recombinant antigen provided high specificity in an i-ELISA but lower sensitivity than the whole bacterial antigen.

The same research team selected rOmp16, rOmp25, rBP26, rOmp2b, and rOmp31 to predict B-lymphocyte epitopes to create a new diagnostic antigen. The paper-based multi-epitope ELISA was evaluated on *Brucella*-positive and *Brucella*-negative bovine and goat sera. It was established that short linear peptides assembled into a single rOmp are also effective antigens for detecting brucellosis-positive sera. The individual proteins detected antibodies in only a part of the sera, whereas the fusion protein identified antibodies in almost all positive sera. Moreover, the multi-epitope protein did not interact with rabbit antibodies against bacteria with a similar antigenic structure to *Brucella* spp. [69].

rBP26, rOmp25, rOmp31, and the multi-epitope fusion protein (rBP26 + rOmp16 + rOmp2b + rOmp25 + rOmp31) were evaluated by i-ELISA for their potential use as antigens in the serological diagnosis of canine brucellosis compared with conventional methods. The multi-epitope antigen best differentiated positive and negative dog sera, with positive (100%) and negative (98%) predictive values. rBP26 and rOmp31 showed superior sensitivity for detecting anti-*Brucella* antibodies in canine sera. However, they interacted with rabbit sera that were infected with *Vibrio parahaemolyticus* and *Listeria monocytogenes*, which may limit their use as antigens. Recombinant Omp25 was characterized by relatively low sensitivity and showed a limited ability to differentiate between positive and negative dog sera [70].

In another study, bioinformatics tools selected linear B-cell epitopes from the rOmp22, rOmp25, and rOmp31 antigens. The antigenicity of the fusion protein was studied on blood serum samples from patients and healthy people by immunoblotting and i-ELISA. The results of immunoblotting, as well as the accuracy, sensitivity, and specificity of i-ELISA based on a multi-epitope protein, showed that this antigen has good prospects in the serodiagnosis of brucellosis [71].

We have created three types of *Brucella* antigens, each consisting of immunodominant regions of two major *Brucella* Omps [72]. They were successfully expressed in *E. coli* BL21 (DE3) cells using the pET28 plasmid and were tested in i-ELISA for the serological diagnosis of cattle and sheep brucellosis. These chimeric proteins, designated as rOmp19 + 25, rOmp19 + 31, and rOmp25 + 31, consisted of active serological regions of *Brucella* proteins with MWs of 19, 25, and 31 kDa [73].

Antibodies against rOmp19 + 31 and rOmp25 + 31 in the blood serum of a hyperimmunized rabbit were detected up to a serum dilution of 1:12800, while the titer of antibodies to single recombinant proteins (rOmp19 and rOmp25) did not exceed 1:1600. For further evaluation of chimeric proteins, sera obtained from unvaccinated (n = 43), naturally (n = 77), and/or experimentally infected cattle (n = 12) were used. Chimeric proteins showed maximum sensitivity with a specificity of 95%–100% and an accuracy of 97%–100%, while these indicators when using individual rOmps were in the range of 88%–97%, 79%–93%, and 73%–87%, respectively. The affinity of antibodies to rOmp19 + 31 was significantly higher than that to individual proteins and to rOmp19 + 25 and rOmp25 + 31. Antibodies against chimeric rOmps appeared in the blood of all experimentally infected cattle on the 14^th^-day post-infection; however, only 67% and 58% of the animals had antibodies to rOmp25 and rOmp31, respectively.

It should be noted that the serological potential of recombinant antigens is being studied only in an i-ELISA. Competitive ELISA (c-ELISA) has several advantages over i-ELISA. First, samples from different species may be tested using a single enzyme-labeled anti-species antibody [74]. Second, c-ELISA is characterized by a higher specificity than i-ELISA and, therefore, is an excellent confirmatory test for the diagnosis of brucellosis in most mammalian species [75, 76]. Third, it can be adapted for DIVA by selecting mAb with the desired affinity [77]. In addition, the diagnostic value of recombinant proteins in serological tests that are easy to perform remains poorly understood. Preliminary data on the use of recombinant periplasmic proteins in the latex agglutination test [78] and immunochromatographic analysis [79, 80] promise encouraging results in the development of portable rapid tests for screening several samples for brucellosis.

## Conclusion

*Brucella* recombinant proteins are being extensively studied for immunogenicity and protectiveness with the aim of creating new generation vaccines. As for the diagnostic value of *Brucella* proteins, it still remains insufficiently studied, and the available data are controversial.

These discrepancies are primarily due to the use of conventional serological tests as a standard, while the “gold standard” (bacteriological analysis) for the correct classification of sera into *Brucella* antibodies positive and negative was used in only a few studies testing a small number of food-producing animals, dogs, and humans. Studies on the serological potential of *Brucella* recombinant proteins in sera from experimentally infected cattle, sheep, and goats have been conducted by only a few scientific groups. Moreover, the obtained results do not allow for drawing unambiguous conclusions. However, the available data suggest that the diagnostic value of a recombinant antigen may vary depending on the species and physiological state of an animal, as well as the *Brucella* spp. and the epidemiological situation of brucellosis in the region. This requires further comprehensive study.

Among cell wall proteins, the periplasmic protein pBP26 is relatively well studied and is the most suitable recombinant protein that can be used as an antigen for the serological diagnosis of human and animal brucellosis.

While BP26 has shown potential as a diagnostic antigen, there are also indications of its low specificity. Thus, for the correct selection of a recombinant protein or its immunoreactive region as a reliable diagnostic antigen, species-specific sera obtained by immunization of food-producing animals and/or dogs with pathogens that have antigenic similarity to *Brucella* spp. are needed.

The evaluation of rSOD and rBP26 for their potential to distinguish vaccinated from naturally infected cattle, using serological tests without a “gold standard” and the results of studying the antigenicity of rBP26 in the sera of a small number of experimentally infected and/or vaccinated sheep are not sufficient to evaluate the prospects of these proteins as antigens for DIVA. Nevertheless, they deserve attention as possible candidates for the development of a new antigen for the reliable diagnosis of brucellosis.

Finally, the possibility of using recombinant proteins in the development of simpler tests suitable for serological testing of animals in poorly equipped laboratories and/or under field or rural conditions remains unexplored. This line of research is highly relevant given that brucellosis tends to be prevalent in developing countries. Therefore, further studies are required to investigate the specificity of *Brucella* spp. recombinant cell wall proteins, not only in relation to host species but also pathogen species, and to explore the potential for developing new diagnostic methods, both in laboratory and lab-free settings, for brucellosis diagnosis.

## Authors’ Contributions

AB: Conducted a comprehensive literature search, analyzed the gathered data, and drafted the manuscript. SE: Conducted the final revision and proofreading of the manuscript. Both authors have read, reviewed, and approved the final manuscript.
